# Periodontal Health Status among Patients with Behçet's Disease

**DOI:** 10.1155/2016/7506041

**Published:** 2016-02-29

**Authors:** Zahra Habibagahi, Hooman Khorshidi, Shahrzad Hekmati

**Affiliations:** ^1^Department of Internal Medicine, School of Medicine, Shiraz University of Medical Sciences, P.O. Box 71345-1836, Shiraz, Iran; ^2^Department of Periodontology, School of Dentistry, Shiraz University of Medical Sciences, P.O. Box 71345-1836, Shiraz, Iran; ^3^School of Medicine, Shiraz University of Medical Sciences, P.O. Box 71345-1836, Shiraz, Iran

## Abstract

We aimed to evaluate the relationship of individual periodontal parameters with the severity of Behçet's disease (BD) and attempt to find the correlation between chronic periodontitis and BD. In this study, 74 registered subjects attending Behçet's clinic with BD symptoms were recruited. The diagnosis was based on the criteria presented by the international study group for BD (ISG) and the total clinical severity score was determined for each patient. All individuals underwent clinical examination to assess oral and periodontal status and presence of oral ulcers. Periodontal clinical parameters of bleeding on probing (BOP), probing depth (PD), and clinical attachment level (CAL) and also hygiene index (HI) and decayed, missing, and filled (DMF) teeth were noted and analyzed to assess the correlation with severity of BD. There was no significant correlation between DMF and severity of BD. The strong association was found between periodontal parameters (BOP, PD, and CAL) and the severity of BD (*P* < 0.001). It seems that relation of BD to oral health is higher up in severe forms of BD and periodontal diseases. Clinical association between the diseases might be due to a common underlying etiopathogenesis of periodontitis and BD.

## 1. Introduction 

Behçet's disease (BD) is a chronic immune-mediated systemic vasculitis, which tends to manifest as mucocutaneous lesions and may affect the gastrointestinal, nervous, and cardiovascular systems. The disease was originally described by a Turkish dermatologist as a triple symptom complex involving the oral, genital, and ocular structures [1–5]. While BD can affect both genders with a wide geographical distribution, it is more prevalent among populations along the “silk route” and the symptoms have been reported to be more severe among young male patients [6–11].

The exact mechanism of the disease remains unknown; however, it is hypothetically said that infectious microorganisms may play a role in setting off the inflammatory response in genetically susceptible patients [9, 12–14]. Oral microbial flora has long been implicated in the pathogenesis, as BD starts mostly from the oral mucosal surfaces. Several evidences indicate that oral microbial flora has a critical role in the pathogenesis of BD [15, 16]. There are no specific diagnostic laboratory tests for BD and diagnosis is made on the basis of clinical finding [17, 18]. Periodontitis is characterized by gingival inflammation accompanied by loss of supportive connective tissues, including the alveolar bone, which subsequently results in the deterioration of the periodontal ligament. Chronic periodontitis is the most common form of periodontitis. It has been known as a major cause of tooth loss throughout the world [19]. Several conditions may give rise to an increased prevalence, incidence, or severity of periodontitis. A recent study have shown that oral health-related quality of life assessments were impaired in patients with BD and associated with disease activity and treatment modalities [20].

Although the periodontal scores were higher in patients with BD compared to healthy subjects in some studies [16, 21, 22], in this study we sought to evaluate the relationship of individual periodontal parameters with BD activity and severity and attempt to find the correlation between periodontal destruction and BD.

## 2. Materials and Methods

A clinical-based cross-sectional study was conducted jointly in the Department of Rheumatology, School of Medicine, and the Department of Periodontology, School of Dentistry, Shiraz University of Medical Science, Shiraz, Iran.

In this study, 74 registered subjects attending the BD clinic with BD symptoms were recruited. Selection criteria for the study group were the diagnosis of BD based on the criteria presented by the international study group for BD (ISG) [23, 24].

The total clinical severity score was determined for each patient as previously described [6, 7]. The symptoms were categorized as mild, moderate, and severe and scored as 1, 2, and 3, respectively. The total score was calculated based on the severity of the symptoms, that is, the sum of 1 point per each mild symptom (oral aphthous stomatitis, genital ulcers, arthralgia, and typical skin lesions such as erythema nodosum, papulopustular lesions, and folliculitis), 2 points each for moderate symptoms (arthritis, deep vein thrombosis of the legs, anterior uveitis, and gastrointestinal involvement), and 3 points each for severe disease manifestations (posterior/panuveitis, retinal vasculitis, arterial thrombosis, neuro-Behçet, and bowel perforation). Patients with a total score of less than 4 were categorized as mild, between 4 and 6 as moderate, and over 6 as severe.

All individuals underwent clinical oral and periodontal examination by a periodontist to assess periodontal status and presence or absence of oral ulcers. Periodontal pocket depth (the distance between the gingival margin and the bottom of the gingival pocket) and clinical attachment level (the distance between the cementoenamel junction and the bottom of the gingival pocket) were measured in 1 mm increments at 6 sites per tooth by using Williams's periodontal probe. Bleeding on probing (BOP) was measured according to Lang et al. [25]. After measuring the PD, the corresponding sites were inspected for the presence or absence of bleeding. The presence of dental plaque at the gingival margin along the mesial, buccal, distal, and lingual aspects was determined as described by Love et al. [26] in the hygiene index (HI, percentage). Decayed, missing, and filled (DMF) teeth were recorded. Recordings have excluded third molars and root fragments.

Data were submitted to the SPSS software version 15. Mean and standard deviations were calculated and subjected to analysis via analysis of variance (ANOVA). Chi-Square test, Tukey post hoc test, and Pearson's correlation analysis were used in the analysis.

## 3. Results 

A total of 74 patients (mean age: 37.39 ± 10.4 years) were studied.  Table 1 summarizes the gender distribution of patients relative to the severity of the disease. Females were more abundant than males. The incidence of periodontitis in patients with severe BD was significantly higher compared to the mild group (*P* < 0.001).

Mean age of the patients in the severe group was significantly higher compared to the other groups (*P* < 0.001). Mean bleeding in probing, mean probing depth, mean clinical attachment level, and also mean duration of BD were also significantly higher among patients with severe BD (*P* < 0.001). The DMFT values among the study population had no significant relationship with the severity of BD (Table 1).

It was further observed that longer duration of the disease was significantly correlated with age, diseases activity, increased PD (>4 mm), BOP, and CAL (*P* < 0.001) (Table 2).

## 4. Discussion 

In this clinical-based cross-sectional study of periodontal status in Behçet's disease, we found a positive correlation between periodontal destruction parameter including clinical attachment loss, probing depth, and bleeding on probing and the severity of BD. The strong association was found between periodontal parameters (BOP, PD, and CAL) and the severity of BD. The increase in these parameters is a reflection of periodontal destruction. Furthermore in a recent study of association between specific clinical parameters of periodontitis and systemic biomarkers, BOP was the only periodontal parameter significantly associated with systemic parameters of CRP, FIB, and WBC [27]. The absence of BOP can be used as a predictor of periodontal stability [25]. This relationship may be reflecting the potential importance of local periodontal inflammation on systemic inflammation.

Numerous studies have documented that periodontal diseases are related to the onset and progression of various systemic disorders such as cardiovascular disease, diabetes mellitus, preterm low birth weight, and osteoporosis. Researchers have hypothesized the etiologic role of periodontitis in the pathogenesis of these systemic illnesses [28]. Chronic infections of periodontal structures in genetically susceptible patients could accelerate BD by promoting a chronic systemic inflammatory status through the release of bacterial products, heat shock proteins, acute-phase reactants, and any other inflammatory mediators [29].

The present study revealed a positive correlation between periodontitis and severity of BD. Our results were in agreement with some other previous studies which reported higher scores of plaque index, periodontal index, sulcus bleeding, and probing depth in BD patients compared to healthy controls [6, 16, 20, 22]. Mumcu et al. have repeatedly shown that the presence of painful oral ulcers in patients with BD affects the oral health-related quality of life among these patients [16, 20, 30, 31]. The results of our study also agree with Arabaci et al. where the periodontal health is compromised in BD patients and the severity of the periodontal disease is directly correlated with the severity of BD [32]. However, Akman et al. failed to find any significant difference in the periodontal health status between patients with BD and healthy subjects [6]. This controversy may be due to other confounding factors affecting the oral health among the two populations. Similar to our results some studies have demonstrated that patients with moderate and severe BD symptoms have higher scores in all clinical periodontal parameters compared to those with milder symptoms [6, 16, 21, 22].

In terms of gender, the majority of studies report a slightly higher male predilection for this disease. It has been stated that the symptoms occur more severely among young male patients [33]. One study in a Turkish population, however, failed to show any significant gender predilection [32] and, in our study, although we did not find a significant relationship between the disease severity and gender, the female population was higher than the male population. These differences may be attributed to the sampling methods among different studies.

Considering the role of microorganisms in the onset and progression of the disease, it is important for BD patients to maintain an optimum level of oral hygiene and minimize the microbial load in the oral cavity. Therefore, clinicians should attempt to control the painful symptoms which may compromise the oral hygiene performance in patients with BD. Administration of topical glucocorticoids, analgesics, and antibiotics and laser therapy are among the most common approaches to control the microbial load and painful symptoms in patients with BD [32, 34–37].

## 5. Conclusion 

It was demonstrated that periodontal health is impaired by BD and is correlated with the severity of the disease. Therefore, proper oral hygiene maintenance should be considered in order to minimize the symptoms among patients with BD. We demonstrated a statistically significant association between periodontitis and BD activity in our patients. Patients with more severe disease have more severe periodontal involvement. BOP is strongly associated with severity of BD. It seems that relation of BD to oral health is higher up in severe forms of BD and periodontal diseases. Both of these two conditions were studied at the same time, and we cannot conclude that periodontal involvement is a preceding factor in BD. Although there is significant overlap with periodontitis and BD activity, we do not believe that poor oral hygiene sufficiently explains the association. This clinical association between the two diseases might be due to a common underlying etiopathogenesis of periodontitis and BD.

## Figures and Tables

**Table 1:** Comparisons of demographic and periodontal characteristics between severity groups.

		Severity			Total	*P*
		Mild (*n* = 40)	Moderate (*n* = 24)	Severe (*n* = 10)		
Age (years)		34.40 ± (10.19)^a^	41.38 ± (9.513)^b^	39.8 ± (11.134)^ab^	37.39 ± (10.497)	

Sex (%)	F	31 (56.4)	19 (34.5)	5 (9.1)	55 (100)	0.165
	M	9 (47.4)	7 (36.8)	3 (15.8)	19 (100)	

Duration (years)		4.88 ± (3.123)^a^	8.21 ± (3.078)^b^	10.7 ± (3.164)^b^	6.76 ± (3.77)	

BOP (%)		18.87 ± (20.53)^a^	38.75 ± (30.813)^b^	44 ± (30.375)^b^	28.71 ± (27.50)	

PD (mm)		1.37 ± (0.63)^a^	2.17 ± (0.45)^b^	2.87 ± (0.51)^b^	1.82 ± (0.76)	

CAL (mm)		1.54 ± (0.94)^a^	2.51 ± (0.898)^b^	3.029 ± (0.836)^b^	2.06 ± (1.07)	

HI (%)		58.62 ± (28.44)	76.66 ± (29.25)^a^	83.5 ± (18.26)^a^	67.83 ± (29.13)	

DMFT		11.15 ± (4.812)^a^	11.15 ± (4.460)^a^	12.37 ± (4.983)^a^	11.28 ± (4.659)	

Quantitative variables were described using mean ± SD and sex was described using frequency (%).

In each row, mean values with at least a common letter in superscript were not statistically significant (Tukey post hoc test).

**Table 2:** Correlations of periodontal parameters with Behçet's disease severity.

	BD's activity	Duration of BD
Probing depths	0.695 (<0.001)	0.594 (<0.001)
CAL	0.621 (<0.001)	0.572 (<0.001)
BOP%	0.429 (<0.001)	0.412 (<0.001)
HI%	0.380 (0.001)	0.398 (<0.001)
DMFT	0.015 (0.901)	0.154 (0.190)

The values in the table are Pearson's correlation (*P* value).

## References

[1] Verity D. H., Wallace G. R., Vaughan R. W., Stanford M. R. (2003). Behçet's disease: from Hippocrates to the third millennium. *British Journal of Ophthalmology*.

[2] Fortune F. (2003). Can you catch Behçet's disease?. *Journal of Laboratory and Clinical Medicine*.

[3] Marshall S. E. (2004). Behçet's disease. *Best Practice & Research Clinical Rheumatology*.

[4] Yapışlar H., Aydogan S., Borlu M., Ascioglu Ö. (2007). Decreased nitric oxide and increased platelet aggregation levels in patients with Behçet's disease. *Thrombosis Research*.

[5] Gürler A., Boyvat A., Türsen Ü. (1997). Clinical manifestations of Behçet's disease: an analysis of 2147 patients. *Yonsei Medical Journal*.

[6] Akman A., Kacaroglu H., Donmez L., Bacanli A., Alpsoy E. (2007). Relationship between periodontal findings and Behçet's disease: a controlled study. *Journal of Clinical Periodontology*.

[7] Habibagahi M., Habibagahi Z., Saidmardani S.-M., Sadeghian F. (2015). No Definite Association between human parvovirus B19 infection and Behçet disease. *Iranian Journal of Medical Sciences*.

[8] Kural-Seyahi E., Fresko I., Seyahi N. (2003). The long-term mortality and morbidity of Behçet syndrome: a 2-decade outcome survey of 387 patients followed at a dedicated center. *Medicine*.

[9] Türsen Ü. (2012). Pathophysiology of the Behçet's disease. *Pathology Research International*.

[10] Bang D. S., Oh S. H., Lee K.-H., Lee E.-S., Lee S. N. (2003). Influence of sex on patients with Behcet’s disease in Korea. *Journal of Korean Medical Science*.

[11] Tursen U., Gurler A., Boyvat A. (2003). Evaluation of clinical findings according to sex in 2313 Turkish patients with Behçet's disease. *International Journal of Dermatology*.

[12] Evereklioglu C. (2005). Current concepts in the etiology and treatment of Behçet disease. *Survey of Ophthalmology*.

[13] Alpsoy E., Yilmaz E., Coşkun M., Savaş A., Yeğin O. (1998). HLA antigens and linkage disequilibrium patterns in Turkish Behcet's patients. *Journal of Dermatology*.

[14] Galeone M., Colucci R., D'Erme A. M., Moretti S., Lotti T. (2012). Potential infectious etiology of Behçet's disease. *Pathology Research International*.

[15] Lehner T. (1997). The role of heat shock protein, microbial and autoimmune agents in the aetiology of Behçet's disease. *International Reviews of Immunology*.

[16] Mumcu G., Ergun T., Inanc N. (2004). Oral health is impaired in Behçet's disease and is associated with disease severity. *Rheumatology*.

[17] Chajek T., Fainaru M. (1975). Behcet's disease. Report of 41 cases and a review of the literature. *Medicine*.

[18] Ataollahi M. R., Aflaki E., Nazarinia M. A. (2012). Anti-cardiolipin and anti-neutrophil cytoplasmic antibodies in Iranian patients with Behcet's disease. *Iranian Journal of Immunology*.

[19] Burt B. (2005). Epidemiology of periodontal disease. *Journal of Periodontology*.

[20] Mumcu G., Inanc N., Ergun T. (2006). Oral health related quality of life is affected by disease activity in Behçet's disease. *Oral Diseases*.

[21] Nakae K., Agata T., Maeda K., Masuda K., Hashimoto T., Inaba G. (1981). *Behcet's Disease, Pathogenic Mechanism and Clinical Future: Case Control Studies on Behcet's Disease*.

[22] Çelenligil-Nazliel H., Kansu E., Ebersole J. L. (1999). Periodontal findings and systemic antibody responses to oral microorganisms in Behçet's disease. *Journal of Periodontology*.

[23] International Study Group for Behçet's Disease (1990). Criteria for diagnosis of Behçet's disease. *The Lancet*.

[24] Davatchi F. (2012). Diagnosis/classification criteria for Behcet's disease. *Pathology Research International*.

[25] Lang N. P., Adler R., Joss A., Nyman S. (1990). Absence of bleeding on probing. An indicator of periodontal stability. *Journal of Clinical Periodontology*.

[26] Love W. D., Ramirez J. M., Fultz R. P. (1975). An oral hygiene measurement system for possible research and clinical use. *Journal of Public Health Dentistry*.

[27] Bokhari S. A., Khan A. A., Butt A. K. (2014). Periodontitis in coronary heart disease patients: strong association between bleeding on probing and systemic biomarkers. *Journal of Clinical Periodontology*.

[28] Kim J., Amar S. (2006). Periodontal disease and systemic conditions: a bidirectional relationship. *Odontology*.

[29] Angeli F., Verdecchia P., Pellegrino C. (2003). Association between periodontal disease and left ventricle mass in essential hypertension. *Hypertension*.

[30] Mumcu G., Niazi S., Stewart J. (2009). Oral health and related quality of life status in patients from UK and Turkey: a comparative study in Behcet's disease. *Journal of Oral Pathology and Medicine*.

[31] Mumcu G., Hayran O., Ozalp D. O. (2007). The assessment of oral health-related quality of life by factor analysis in patients with Behcet's disease and recurrent aphthous stomatitis. *Journal of Oral Pathology and Medicine*.

[32] Arabaci T., Kara C., Çiçek Y. (2009). Relationship between periodontal parameters and Behçet's disease and evaluation of different treatments for oral recurrent aphthous stomatitis. *Journal of Periodontal Research*.

[33] Mendes D., Correia M., Barbedo M. (2009). Behçet's disease—a contemporary review. *Journal of Autoimmunity*.

[34] Liu J., Zeng X., Chen Q. (2006). An evaluation on the efficacy and safety of amlexanox oral adhesive tablets in the treatment of recurrent minor aphthous ulceration in a Chinese cohort: a randomized, double-blind, vehicle-controlled, unparallel multicenter clinical trial. *Oral Surgery, Oral Medicine, Oral Pathology, Oral Radiology and Endodontology*.

[35] Altenburg A., El-Haj N., Micheli C., Puttkammer M., Abdel-Naser M. B., Zouboulis C. C. (2014). The treatment of chronic recurrent oral aphthous ulcers. *Deutsches Arzteblatt International*.

[36] Fani M. M., Ebrahimi H., Pourshahidi S., Aflaki E., Shafiee Sarvestani S. (2012). Comparing the effect of phenytoin syrup and triamcinolone acetonide ointment on aphthous ulcers in patients with Behcet's syndrome. *Iranian Red Crescent Medical Journal*.

[37] Zand N., Ataie-Fashtami L., Djavid G. E. (2009). Relieving pain in minor aphthous stomatitis by a single session of non-thermal carbon dioxide laser irradiation. *Lasers in Medical Science*.

